# The Role of the Melatoninergic System in Circadian and Seasonal Rhythms—Insights From Different Mouse Strains

**DOI:** 10.3389/fphys.2022.883637

**Published:** 2022-04-12

**Authors:** Martina Pfeffer, Charlotte von Gall, Helmut Wicht, Horst-Werner Korf

**Affiliations:** ^1^ Institute of Anatomy II, Medical Faculty, Heinrich Heine University, Düsseldorf, Germany; ^2^ Dr. Senckenbergische Anatomie II, Fachbereich Medizin der Goethe-Universität, Frankfurt am Main, Germany; ^3^ Institute of Anatomy I, Medical Faculty, Heinrich Heine University, Düsseldorf, Germany

**Keywords:** melatonin, jet lag, activity rhythms, mouse strains, seasonality, chronobiology

## Abstract

The melatoninergic system comprises the neurohormone melatonin and its molecular targets. The major source of melatonin is the pineal organ where melatonin is rhythmically produced during darkness. In mammals, melatonin biosynthesis is controlled by the central circadian rhythm generator in the suprachiasmatic nucleus (SCN) and photoreceptors in the retina. Melatonin elicits its function principally through two specific receptors called MT1 and MT2. MT1 is highly expressed in the SCN and the hypophysial pars tuberalis (PT), an important interface for control of seasonal functions. The expression of the MT2 is more widespread. The role of the melatoninergic system in the control of seasonal functions, such as reproduction, has been known for more than 4 decades, but investigations on its impact on the circadian system under normal (entrained) conditions started 2 decades later by comparing mouse strains with a fully functional melatoninergic system with mouse strains which either produce insufficient amounts of melatonin or lack the melatonin receptors MT1 and MT2. These studies revealed that an intact melatoninergic system is not required for the generation or maintenance of rhythmic behavior under physiological entrained conditions. As shown by jet lag experiments, the melatoninergic system facilitated faster re-entrainment of locomotor activity accompanied by a more rapid adaptation of the molecular clock work in the SCN. This action depended on MT2. Further studies indicated that the endogenous melatoninergic system stabilizes the locomotor activity under entrained conditions. Notably, these effects of the endogenous melatoninergic system are subtle, suggesting that other signals such as corticosterone or temperature contribute to the synchronization of locomotor activity. Outdoor experiments lasting for a whole year indicate a seasonal plasticity of the chronotype which depends on the melatoninergic system. The comparison between mice with an intact or a compromised melatoninergic system also points toward an impact of this system on sleep, memory and metabolism.

## Introduction

**FIGURE 1:**
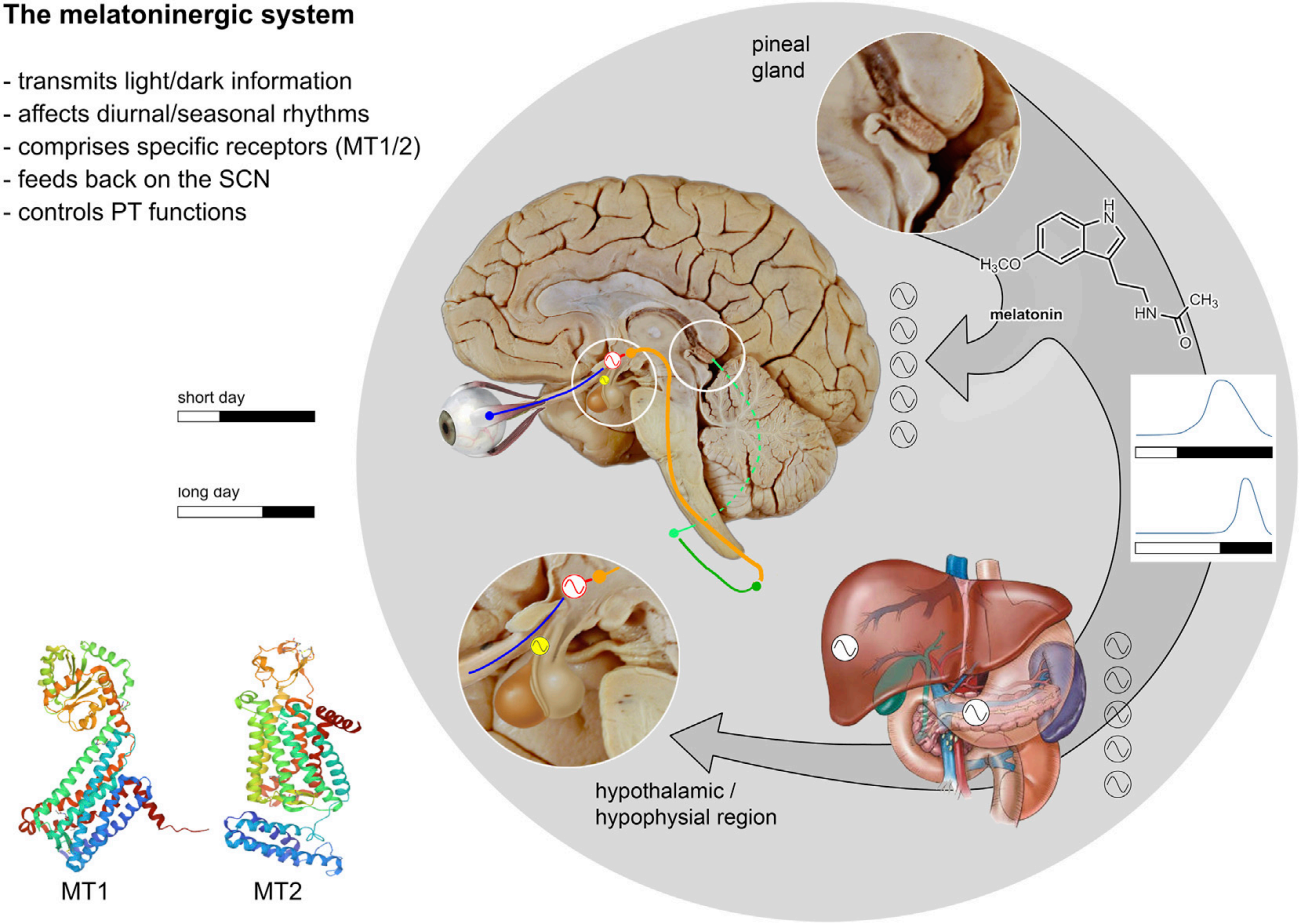
Relationships between the melatoninergic system and the circadian system. The melatoninergic system comprises the neurohormone melatonin and its molecular targets and is closely connected to the circadian system. The core of the circadian system is located in the hypothalamic SCN (red sinus curve) producing a self-sustained endogenous rhythm with a period length of approximately 24 h. This circadian rhythm generated in the SCN is entrained to the ambient light/dark cycle- which varies with time of day or season (see diagrams). Light/dark signals are received by melanopsinergic ganglion cells of the retina and transmitted to the SCN via the retino-hypothalamic tract (blue). The SCN sends efferent projections to the adjoining paraventricular nucleus (shown in orange), which is the main source of descending autonomic projections to the spinal cord. The pineal gland, the major source of the neurohormone melatonin, is controlled by the sympathetic innervation comprising the intermediolateral column of the thoracic spinal cord (dark green) and the superior cervical ganglion (light green). The pineal gland synthesizes and secrets melatonin at night under the control of the SCN. Thus, melatonin represents an output signal of the circadian system. The length of the melatonin signal corresponds with the length of the dark phase (see light/dark diagrams). Via the blood stream, melatonin provides a humoral signal synchronizing various peripheral oscillators (black and white sinus curves) with the day/night rhythm. The main molecular targets of melatonin are the two melatonin receptors: MT1 and MT2, which are located throughout the body and brain. Within the brain, the highest MT-receptor densities are found in the SCN. Thus, melatonin is not only an output signal, but also an input signal to the SCN. The pars tuberalis of the hypophysis (PT, yellow) is another region with high MT-receptor densities. The oscillatory processes in the PT critically depend on the melatonin signal. These oscillations are of importance with respect to the maintenance of seasonal rhythms (after Pfeffer et al., 2018; MT1 structure: Stauch et al., 2019; MT2 structure: Johansson et al., 2019).

Since the origin of life, organisms are influenced by the cyclic lighting conditions of their environment which generate daily/diurnal and seasonal rhythms. In vertebrates the melatoninergic system plays an important role for transmission of light/dark signals and thereby modulates rhythmic behavior. The melatoninergic system comprises the neurohormone melatonin, an indoleamine, and its molecular targets (Figure 1). The main source of melatonin is the pineal organ which produces melatonin during the nighttime and secretes it into the general circulation or into the cerebrospinal fluid. This rhythm is a common feature of all vertebrates, irrespective of whether they are active during the day or the night. Melatonin was isolated by Lerner and colleagues (Lerner et al., 1958, 1960) and identified as the agent responsible for pigment aggregation in amphibian melanophores (see Rollag, 1988). The main steps of melatonin biosynthesis were discovered soon after the isolation of melatonin. According to current concepts the highly lipophilic melatonin is not stored within the pineal organ but released into the bloodstream or the cerebrospinal fluid immediately after its formation. Therefore, the amount of circulating melatonin solely depends on the activity of its biosynthetic pathway. Notably, the penultimate enzyme of the melatonin bioynthesis, the arylalkylamine N-acetyltransferase (AA-NAT) controls daily changes in melatonin production by the pineal gland and thereby plays a unique role in biological timing in vertebrates (Klein, 2007).

In mammals, the rhythmic secretion of melatonin from the pineal gland is driven by the suprachiasmatic nucleus (SCN) of the hypothalamus, the core of the circadian system that generates a circadian rhythm with a period of approximately, but not precisely 24 h and controls many behavioral and physiological functions like rest/sleep, body temperature and hormone secretion. The SCN controls not only the melatonin biosynthesis in the pineal but also many other organs, such as the adrenal and the liver (Korf and von Gall, 2012, 2016; Hastings et al., 2014). The generation of circadian rhythms depends on a molecular clockwork that runs in all nucleated cells and consists of an autoregulatory transcriptional/translational feedback loops of clock genes, particularly *Per1–2, Clock, Bmal1, Cry1–2* (Reppert and Weaver, 2002).

Under natural conditions, the phase and the period length of the rhythm is influenced by environmental stimuli, so called *zeitgebers* (Aschoff and Wever, 1962) in order to adjust (“entrain”) the endogenous clock to the rhythmic events in the outside world (Korf and von Gall, 2012). Light—which varies with time of day or season—is the most potent entraining signal. In non-mammalian vertebrates, light stimuli are perceived by multiple photoreceptors located outside the retina, e.g., in the pineal organ and so-called deep brain (encephalic) photoreceptors (Korf et al., 1998; Nakane and Yoshimura, 2019).

In mammals, the pineal organ has lost its photoreceptive function and the light signals entraining the circadian system are exclusively perceived in the retina by classic and specific nonvisual photoreceptors (Peirson et al., 2018) and are transmitted via the retinohypothalamic tract to SCN in order to synchronize the endogenous clock in the SCN with the light/dark phase. The SCN conveys its output signals to the periphery via endocrine and multisynaptic neural pathways involving the sympathetic and parasympathetic nervous system (Buijs and Kalsbeek, 2001). The sympathetic nervous system is essential for the control of melatonin biosynthesis in the mammalian pineal organ. Postganglionic nerve fibers originating from the superior cervical ganglion (Kappers, 1964; Korf al. al., 1998) release norepinephrine (NE) at night which stimulates melatonin synthesis (Klein and Weller, 1970). The signal transduction cascades activated by NE involve cyclic AMP and transcriptional and posttranscriptional mechanisms, they vary from one mammalian species to the other (Schomerus and Korf, 2005). The robust nightly peak of melatonin secretion is an important neuroendocrine output signal of the circadian system, which reflects the length of the dark period and transduces photoperiodic information (Pevet, 2002; Korf, 2018). Melatonin in turn modulates the rhythm of the SCN and exogenously administered melatonin and its agonists are used as chronobiotics to treat rhythm disturbances/misalignments occurring in blind people, shift workers and after jet lag (Arendt et al., 1997; Arendt, 2009; Arendt, 2010; Liu et al., 2016).

In addition to the pineal organ melatonin can also be produced in the retina (rhythmic; for review see Tosini et al., 2012) and in various cells of the gastrointestinal tract and the immune system (non-rhythmic; for review see Bubenik, 2002; Markus et al., 2018). However, in these structures melatonin rather act as a local modulator and do not contribute to melatonin levels in the blood or cerebrospinal fluid.

### Melatonin Receptors

Targets of melatonin have been first identified as iodomelatonin binding sites by (Vanĕcek et al., 1987) and later on by Williams (1989). It is now well established that melatonin exerts its physiological effects principally through two membrane bound high affinity G-protein coupled receptors which have been cloned by Reppert and colleagues, initially termed as Mel1a and Mel1b (Reppert et al., 1994; Reppert et al., 1995) and now called MT1 and MT2. While the MT1 receptor is concentrated in the SCN and the pars tuberalis of the hypophysis (PT), the MT2 receptor is more widespread and found in several brain areas and throughout the body (Dubocovich, 2007; Slominski et al., 2012; Jockers et al., 2016; Cecon et al., 2018). This has been confirmed by a recent study in mice by means of a “knock-in” strategy replacing MT1 or MT2 coding sequences with a LacZ reporter (Klosen et al., 2019). Expression of MT1 was shown in very few structures such as the SCN and the PT, while expression of MT2 was not only found in the SCN and the PT, but also in numerous other brain regions including the olfactory bulb, forebrain, hippocampus, amygdala and superior colliculus. However, a study using a MT1 transgenic reporter mouse, suggests that MT1 is also expressed in many parts of the brain including the cerebellum, the hippocampus, and the habenula (Adamah-Biassi et al., 2014). Even in those regions where co-expression of the two subtypes was observed MT1 and MT2 were expressed by different cell types. The two receptor subtypes also differ in terms of affinity and the second messenger pathways involved (Jockers et al., 2016; Cecon et al., 2018). Both receptors may dimerize to form homo- or heterodimers among themselves (Ayoub et al., 2002; Baba et al., 2013; Ferré et al., 2014; Cecon et al., 2018). In addition, the orphan receptor, GPR 50, which does not bind melatonin is a dimerization partner for MT1, and this heterodimerization appears to inhibit the MT1 activity (Levoye et al., 2006; Levoye et al., 2006).

*In vitro* studies revealed that both receptors serve different functions in the SCN. The MT1 receptor mediates the acute inhibition of the electrical activity of SCN neurons (Liu et al., 1997) and of PACAP-mediated signal transduction (Jin et al., 2003; von Gall et al., 1998; von Gall et al., 2000). The MT2 receptor has been shown to mediate the phase-shifting effects of melatonin on neuronal firing in SCN cultures of rats (McArthur et al., 1997; Hunt et al., 2001) and mice (Dubocovich et al., 1998; Dubocovich et al., 2005; Korf and von Gall, 2006).

In addition to MT1 and MT2 melatonin may act on the quinone reductase QR2 (Nosjean et al., 2000) and might be involved in mediating the antioxidant effects of melatonin (Reiter et al., 2000). In addition, due to its lipophilic nature, melatonin passes the cell membrane and might interact directly with intracellular proteins (Benítez-King, 2006). Thus, melatonin receptor-independent responses might also play a role in melatonin-dependent mechanisms (Reiter et al., 2014). However, the role of these melatonin targets is still unclear.

### Mouse Models for Investigations of the Impact of the Melatoninergic System Under Physiological Conditions

**FIGURE 2:**
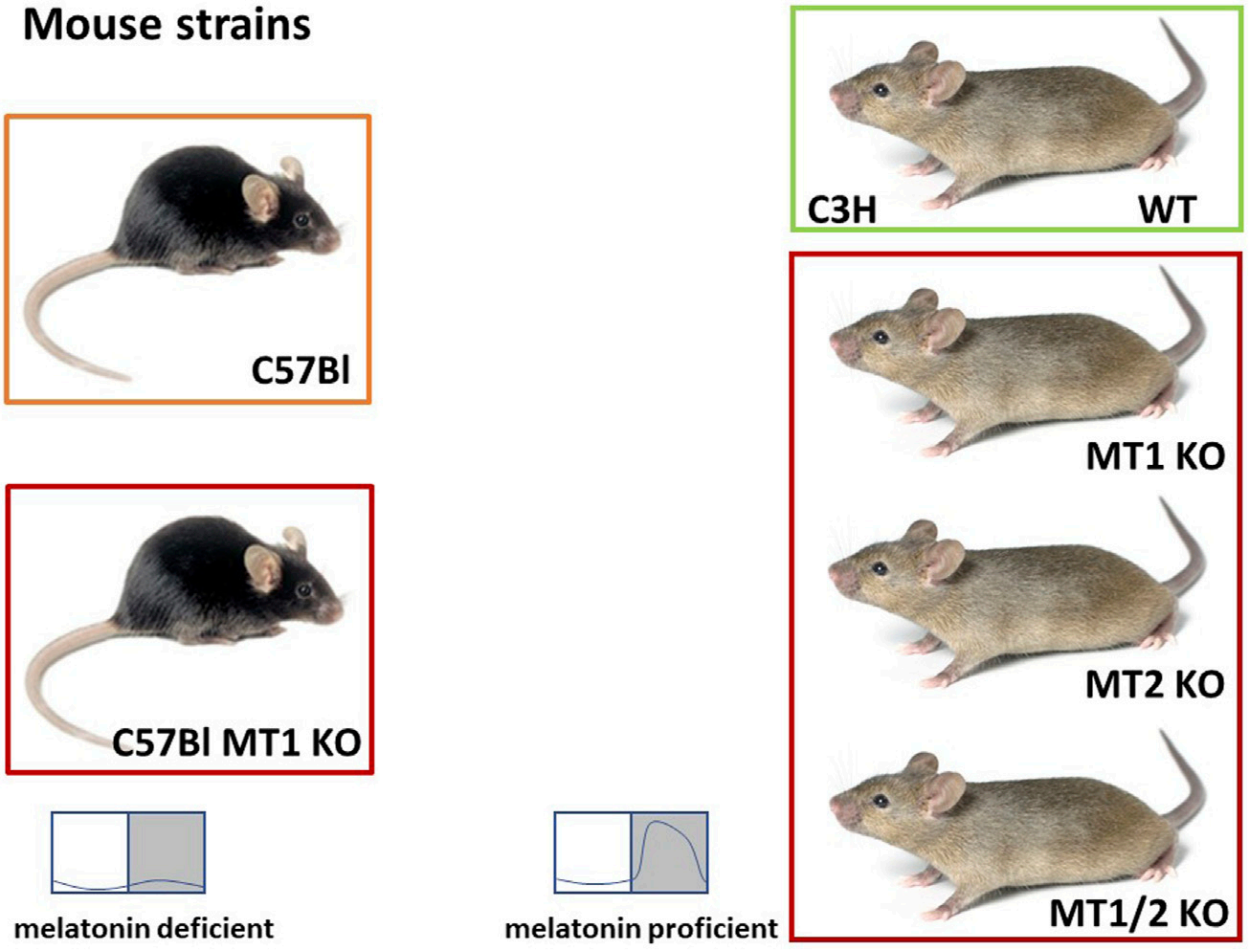
Overview of the mouse models used to elucidate the role of melatonin and the melatonin receptors (MT1/2). The C57Black/6J (C57Bl) mice are melatonin deficient. C57Bl mice with a targeted deletion of the MT1 gene (C57Bl MT1 KO; Liu et al., 1997) were initially used for experiments elucidating the role of externally applied melatonin. C3H/HeN (C3H) mice are melatonin-proficient, but visually blind. C3H mice with a targeted deletion of the MT1 receptor (C3H MT1 KO) or the MT2 receptor (C3H MT2 KO) were obtained by breeding the initial melatonin receptor KO mice (Liu et al., 1997; Jin et al., 2003) on a melatonin-proficient C3H/HeN background for at least 10 generations. C3H double melatonin receptor deficient mice (C3H MT1/2 KO) were obtained by crossing C3H MT1 KO and C3H MT2 KO mice and breeding the MT1/2 KO offspring for at least 10 generations. These animals are used to investigate the role of endogenous melatonin and the role of the melatonin receptors (C3H and C57Bl mouse: Jax.org).

Externally applied melatonin is successfully used as a chronobiotic drug to treat desynchronization and circadian disorders, and the success of these treatments suggest a pivotal role of melatonin in the synchronization of the circadian system. Investigations with various mouse strains with an intact or a compromised melatoninergic system have contributed to decipher its role of under physiological, i.e., entrained conditions. The first experiments compared two mouse strains, C57BL/6J (C57Bl) and C3H/HeN (C3H) (Korf and von Gall, 2006; Figure 2). C57Bl is the classical strain to generate transgenic mice and is widely used in several research fields. Like many inbred mouse strains, the C57Bl are melatonin-deficient due to a spontaneous mutation in a gene encoding for the AA-NAT, the key enzyme of melatonin biosynthesis (Roseboom et al., 1998). C57Bl mice produce only very low amounts of melatonin in the pineal gland and have barely detectable melatonin levels in the circulation which do not show a day/night rhythm (Ebihara et al., 1986; Goto et al., 1989; von Gall et al., 2000). C57Bl mice express both melatonin receptors in different areas of the brain and the retina (Siuciak et al., 1990; Sengupta et al., 2011). Importantly, the signal transduction pathways downstream of these receptors are intact within the SCN and pineal organ of these mice (von Gall et al., 1998; von Gall et al., 2000).

C3H mice are melatonin-proficient and produce high levels of melatonin in the pineal gland at night (Vivien-Roels et al., 1998; von Gall et al., 2000). However, this strain carries a mutation in the retinal degeneration (rd) gene which makes them visually blind 6 weeks after birth (Kim et al., 2008), but they still own the melanopsin containing ganglion cells which are sufficient to mediate the entrainment of the circadian system to light (Peirson et al., 2018).

Of course, the C57Bl mice differ from C3H mice not only with regard to the melatoninergic system, but in many more respects (Kaku et al., 1988; Brednow and Korf, 1998; Veasey et al., 2000). Thus, not every difference between these two strains can be attributed to the melatoninergic system. Therefore, C57Bl mice with targeted deletions of either the MT1 or MT2 or both receptors (Liu et al., 1997; Jin et al., 2003) were back-crossed on a melatonin-proficient C3H background. These melatonin receptor KO, but melatonin-proficient mice were used to study the role of the receptors in melatoninergic signaling (Figure 2). These melatonin receptor KO mice do show some peculiarities that resemble melatonin-deficient C57Bl mice—and in these cases it is likely that the melatoninergic system is responsible (see below).

Recently two “congenic C57BL/6 strains” with a functional melatonin synthesis were developed (Zhang et al., 2018; Zhang et al., 2021), which can be used to investigate the role of endogenous melatonin within one strain.

### Locomotor Output and Entrainment

The locomotor activity is an excellent marker for the state of the circadian system (Shimomura et al., 2001; Refinetti, 2010; van Oosterhout et al., 2012). To study the impact of the melatoninergic system on circadian rhythm generation several investigations compared the locomotor activity rhythms of melatonin-proficient C3H mice with that of melatonin-deficient C57Bl mice (Shimomura et al., 2001; von Gall et al., 1998; von Gall et al., 2000; Pfeffer et al., 2012; Adamah-Biassi et al., 2013; Wicht et al., 2014; Pfeffer et al., 2017).

Both melatonin-proficient C3H and melatonin-deficient C57Bl mice entrain their locomotor activity rhythm to a light/dark cycle and show a rhythmic behavior under free-running conditions; i.e., constant darkness (Kasahara et al., 2010; von Gall et al., 1998; Pfeffer et al., 2012; Wicht et al., 2014). Endogenous melatonin also had no effect on free-running wheel behavior in constant darkness and spontaneous homecage behaviors in melatonin-proficient congenic C57BL/6 line as compared to their melatonin-deficient littermates (Zhang et al., 2021). Therefore, an intact melatonin biosynthesis is not required for the generation or maintenance of rhythmic behavior.

However, detailed and refined analyses have allowed to determine strain-specific differences in the rhythm characteristics of locomotor activity. In melatonin-proficient C3H mice the spontaneous locomotor activity occurs predominantly in the first half of the dark phase (Pfeffer et al., 2012; Adamah-Biassi et al., 2013; Wicht et al., 2014) and declines thereafter. This decline coincides with the nightly peak levels of endogenous melatonin (Maronde et al., 1999; von Gall et al., 2000; Christ et al., 2010). In melatonin-deficient C57Bl mice, the locomotor activity is distributed across the entire dark phase or displays a bi-phasic profile (Pfeffer et al., 2012; Adamah-Biassi et al., 2013; Wicht et al., 2014; Pfeffer et al., 2017). Notably, melatonin receptor KO mice kept a monophasic activity pattern, characteristic for C3H animals (Pfeffer et al., 2017), suggesting that the melatoninergic system is not involved in the constitution of this particular pattern. This assumption is confirmed by the observation that the congenic melatonin-proficient C57BL/6 mouse line keeps the biphasic activity pattern (Zhang et al., 2021), characteristic for C57Bl mice.

C3H and C57Bl differ in terms of the light-induced phase shift in locomotor activity: When a light pulse is given during the second half of the subjective night (when melatonin levels are elevated in C3H, but not in melatonin-deficient C57Bl mice), the phase shifts are smaller in C3H as compared to C57Bl (von Gall et al., 1998). In addition, jet lag experiments (phase delay and phase advance by 6 h respectively) have shown that the melatonin-proficient C3H re-entrain almost twice as fast as the melatonin-deficient C57Bl after a 6 h phase advance (Pfeffer et al., 2012). In an independent study exogenous melatonin has been shown to decrease the number of days necessary for re-entrainment of the spontaneous behaviors in C57Bl mice (Adamah-Biassi et al., 2013) indicating a role of the melatoninergic system in re-entrainment. Indeed, the faster re-entrainment can be directly attributed to the melatoninergic system since in the recently developed melatonin-proficient C57BL/6J congenic mice, re-entrainment of wheel-running activity was accelerated following a 6-h phase advance when compared with their melatonin-deficient littermates (Zhang et al., 2021).

Furthermore, jet lag experiments (phase delay and phase advance by 6 h respectively) with melatonin-proficient C3H mice that either lack the MT1, MT2 or both receptors, have confirmed that melatonin facilitates re-entrainment of the locomotor activity. In C3H, this action depends on the MT2 receptor. The faster re-entrainment to the abrupt advance of dark onset persisted in the MT1 KO animals, but was lost in MT2 KO and double KO animals. However, in an experiment that administered exogenous melatonin to melatonin deficient-C57Bl mice (Dubocovich et al., 2005), an involvement of the MT1 receptor in melatonin-mediated phase shifts of wheel running activity rhythms was observed. These inconsistent results might be explained by different effects of endogenous and exogenous melatonin and/or different periods of sensitivity, desensitization and/or internalization of melatonin receptors. On the other hand, phase shifts may be modulated by the MT1 receptor in C57Bl mice. Therefore, an MT1 receptor mediated effect on phase shifting under certain circumstances cannot be excluded.

The re-entrainment of locomotor activity rhythms is associated with readjustment of the molecular clockwork within the SCN (Reddy et al., 2002; Yamazaki et al., 2000). The above-mentioned jet lag experiments revealed that melatonin-proficient C3H mice with a functional MT2 receptor showed not only faster re-entrainment of the locomotor activity rhythm to the new light/dark cycle, but also a more rapid adaptation of PER1 and CRY1 proteins in the SCN (Pfeffer et al., 2012). These findings provide evidence that melatonin can influence the clock gene expression in the SCN.

### Chronotype/Entrainability/Rhythm Stability

Humans and animals have preferred phase angles of entrainment with regard to external time (Aschoff and Wever, 1962; Ehret, 1974; Roenneberg et al., 2003; Wicht et al., 2014). These are referred as early and late chronotypes (Ehret, 1974). In humans the chronotype is usually defined by the sleep-wake cycle and makes reference to the middle of the sleep and to the timing of the nightly melatonin secretion (Kantermann et al., 2015), and therefore it is tempting to speculate that the melatoninergic system might also play a role in the constitution of the chronotype. At variance with humans, mice as nocturnal animals have the peak of their locomotor activity during night when melatonin levels are high. To identify the chronotype in mice we have introduced as parameter the locomotor activity rather than the sleep-wake cycle (Wicht et al., 2014). Locomotor activity was recorded by means of an infrared camera for at least 10 days and the chronotypes of mice were identified as the timepoint when the animals have performed half of their locomotor activity during a period of 24 h. These measurements indeed showed that the melatonin-deficient C57Bl mice do have a later chronotype in comparison to melatonin-proficient mice C3H (Wicht et al., 2014; Pfeffer et al., 2015; Metzger et al., 2020). But further experiments showed that the chronotype of melatonin-proficient C3H mice was not affected by deletion of the MT-receptors. The MT1-, MT2-, and MT1,2 KO mice maintained the same chronotype as their parent strains (Pfeffer et al., 2017). Therefore, these differences in the chronotype cannot be attributed to MT1- or MT2-dependent signaling. Furthermore, interbreeding experiments with melatonin pro- and deficient mice showed that the chronotype may depend on the genetic background, since the chronotypes of the offspring were “intermediate” between the parent strains (Wicht et al., 2014). Several genetic loci have been identified in mice that affect the period and the phase angle of locomotor behavior (Hofstetter et al., 2003; Wisor et al., 2007) but none of these loci is related to the melatoninergic system. Therefore, it is unlikely that the melatoninergic system plays a pivotal role for determination of the chronotype.

Notably the chronotype in humans changes with the seasons. Short days (in winter) shift the average chronotype of a population to a later chronotype, while long days (in summer) shift it to an earlier one (Allebrandt et al., 2014; Shawa et al., 2018). This seasonal plasticity of the chronotype might be influenced by the melatoninergic system since melatonin secretion is prolonged in winter, when nights are longer as compared to summer, when the nights are shorter (Illnerova et al., 1985; Pevet, 2003).

**FIGURE 3:**
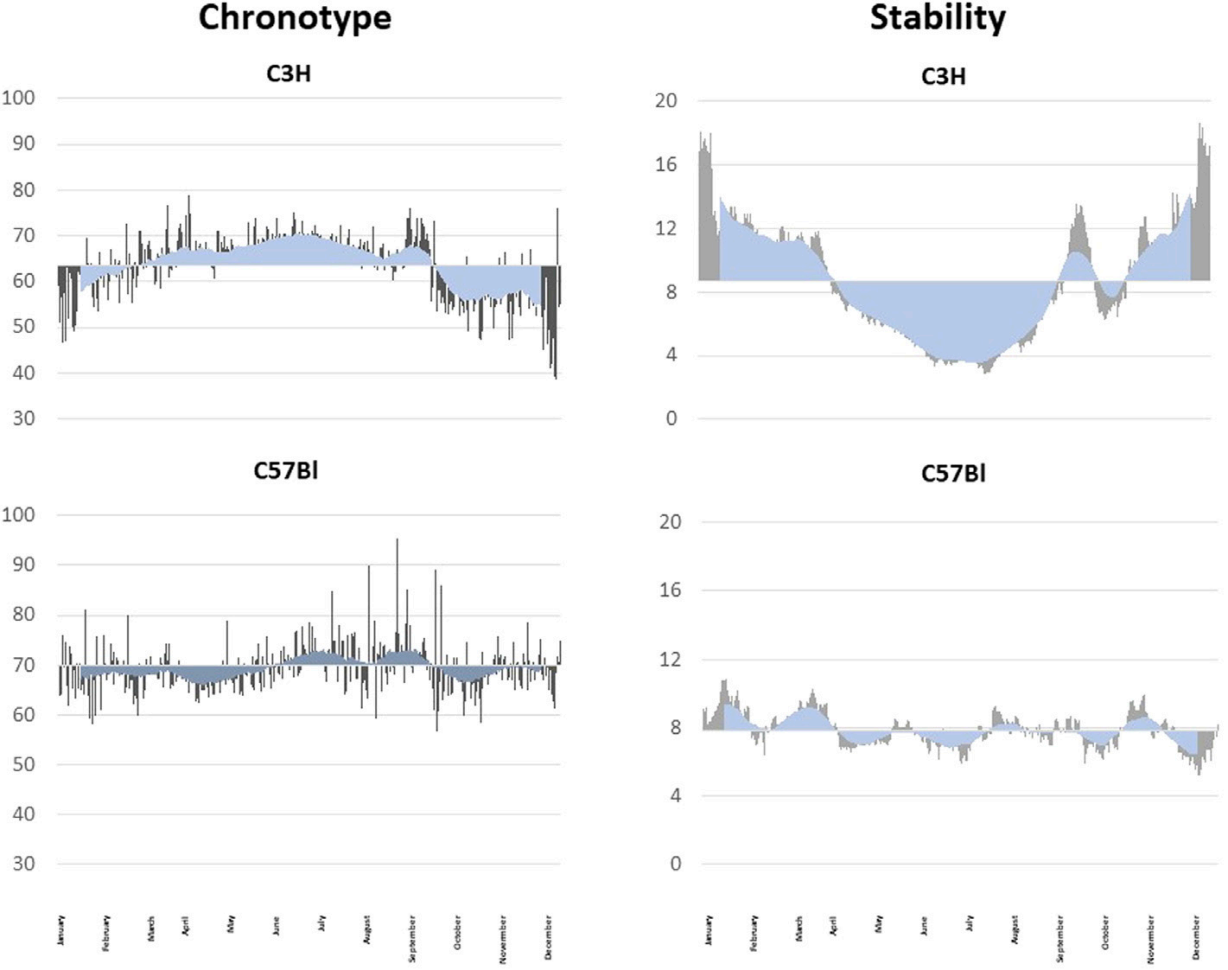
Seasonal fluctuation of the chronotype and the rhythm stability. Analyses of seasonal fluctuations of the daily chronotype and the rhythm stability of a C3H (upper graphs) and a C57Bl mouse (lower graphs) under semi-natural conditions for 1 year. The daily measurements are shown as grey lines, the blue lines indicate the rolling average over 30 days. The data are blotted against the yearly mean. The melatonin-proficient C3H mouse show a clear seasonal difference in the chronotype with a later, more stable chronotype in summer and an earlier, less stable chronotype in winter. The melatonin-deficient C57Bl mice did not show pronounced seasonal changes in the chronotype and has variable stable and unstable periods.

Experiments with C3H and C57Bl mice under seminatural conditions support the assumption that the seasonal plasticity of the chronotype might depend on an intact melatoninergic system. The C3H had a later chronotype in summer and an earlier chronotype in winter. These seasonal changes of the chronotype are far less pronounced in C57Bl mice (Metzger et al., 2020; Figure 3). The timing of the behavior of the melatonin-proficient C3H mice is linked more tightly to the entraining factors (light and night-time temperature) than that of the melatonin-deficient C57Bl strain. It seems that these two mouse strains differ in terms of their capacity to entrain to these stimuli. These differences in the “entrainability” of the two strains were also evident in the jet lag experiments mentioned above as well as in terms of the light-induced phase shift in locomotor activity (von Gall et al., 1998; Pfeffer et al., 2012) indicating a modulating role of melatonin.

One function of the endogenous melatoninergic system might be to stabilize behavioral rhythms under entrained conditions. Indeed, melatonin deficiency or the deletion of both melatonin receptors has been shown to decrease the stability of activity rhythms in mice as compared to mice with an intact melatoninergic system (Pfeffer et al., 2017). Also, the daytime activity has been shown to be slightly higher in melatonin-deficient C57Bl mice as compared to melatonin-proficient C3H mice and melatonin-proficient C3H mice that lack both MT receptor subtypes (Fischer et al., 2017; Homola et al., 2016). However, under semi-natural conditions the activity rhythms of the melatonin-deficient C57Bl mice appeared more stable as compared to the melatonin-proficient C3H animals (Metzger et al., 2020; Figure 3). The C3H mice changed the stability of their activity rhythms with the seasons and, again, C57Bl animals did not show the seasonal changes of the stability (Figure 3). Actually, they appeared more stable as compared to C3H mice (Metzger et al., 2020). But this greater stability may be due to the lesser entrainment to the ambient light/dark cycle and nighttime temperature (Metzger et al., 2020).

Furthermore, the above-mentioned experiments under semi-natural conditions revealed that melatonin-deficient C57BL mice are more vulnerable to changing variables under more natural conditions, which might overexcite the entrainability of these mice. The stabilizing factor may be melatonin, since these destabilized activity rhythms did not occur in the melatonin-proficient C3H animals (Metzger et al., 2020). This assumption is supported by a study by Adamah-Biassi et al. (2014) which shows that melatonin signaling via the MT1 receptor plays a role in maintaining the integrity of spontaneous behavioral rhythms.

### Seasonal Effects

The importance of the melatonin signal was first described in studies of seasonal breeding animals which showed that the duration of the melatonin signal controls the reproductive activity (Reiter, 1991). It is now well established that the seasonally changing melatonin signal is decoded in the hypophysial pars tuberalis (PT) which acts upon an intrinsic thyroid hormone system in the hypothalamus via a retrograde pathway involving PT-specific TSH as a signal (Ono et al., 2008; Yasuo and Korf, 2011; Korf, 2018). Although reproduction is not seasonally regulated in most mouse strains, a strong photoperiodic response was induced in the PT and the intrinsic thyroid hormone in the hypothalamus of melatonin-proficient mice, while this was lacking in melatonin-deficient mice (Ono et al., 2008). These data provided clear evidence that the comparison between melatonin-proficient and -deficient mice is useful to dissect mechanisms through which the melatoninergic system has an effect on seasonal rhythms.

The MT1 receptor has been shown to control the rhythmic expression of the clock gene Per1 (von Gall et al., 2002) as well as the rhythmic expression of several other clock genes (Per2, Bmal1, and Cry1) in the mouse PT (Jilg et al., 2005). MT1 signaling is crucial for the photoperiodic response in the PT and hypothalamus (Yasuo et al., 2009).

### Retina

Also, vision is a rhythmic function adapted to the changes in the daily light/dark cycle and it has been shown that melatonin can modulate a wide variety of retinal functions (for review see Tosini et al., 2012; Felder-Schmittbuhl et al., 2018).

As mentioned above, the retina is capable of rhythmically synthesizing melatonin and contains a molecular clockwork (Tosini and Menaker, 1998; Iuvone et al., 2005; Tosini et al., 2007). Also both melatonin receptors, the MT1 and MT2, have been identified in the layers of the neural retina and in the retinal pigmented epithelium (Bouvier et al., 2009; Baba et al., 2013). A comparison between C57BL and C3H mice showed that in C3H animals, protein levels of PER1 and CRY2 followed a clear day/night rhythm in the inner nuclear layer and the ganglion cell layer with a peak at the end of the day (ZT14) whereas in C57Bl mice protein levels of PER1 and CRY2 did not show significant changes over a 16L/8D cycle (Dinet et al., 2007). Already these data suggest that melatonin may influence PER1 and CRY2 protein levels. Follow-up experiments with C3H and MT1- and MT1,2 KO mice showed that the rhythm in PER1 and CRY2 persisted in the KO mice but the maxima and minima of PER1 were 180° out of phase as compared to C3H animals. These data suggest that the melatoninergic system is not necessary to maintain rhythmic changes in clock-gene protein levels in the murine retina, but appears to be involved in internal synchronization (Dinet and Korf, 2007).

Melatonin is involved in many important retinal functions: it can modulate visual functions and the removal of either MT receptor abolishes the daily rhythms in the scotopic and photopic electroretinograms (Bouvier et al., 2009; Alcantara-Contreras et al., 2011; Sengupta et al., 2011). Melatonin may also have protective effects on retinal cells, since the removal of melatonin receptors affects the viability of the photoreceptors and retinal ganglion cells (Bouvier et al., 2009; Alcantara-Contreras et al., 2011; Gianesini et al., 2016). This suggest that melatonin might be involved in the pathogenesis of age-related macular degeneration and glaucoma.

### Effects on Sleep

The melatonin rhythm is closely associated with the timing of sleep and sleep propensity in humans (Arendt and Skene, 2005). Melatonin has a sleep promoting effect in both (diurnal) humans and (nocturnal) rodents (Holmes and Sugden, 1982; Arendt et al., 1984), and also sleep quality improves when the circadian system is in phase with melatonin rhythm (Morris et al., 2012). However, the sleep-wake cycles of mice differ from those of humans. In contrast to the long sleep period in humans, mice have numerous, short periods of sleep throughout the 24-h cycle (Mitler et al., 1977; Daszuta et al., 1983; Veasey et al., 2000). Nevertheless, mice with an intact or compromised melatoninergic system have been used to investigate the role of melatonin and its receptors in the regulation of the sleep/wake cycle. Unsurprisingly, the typical strain specific daily locomotor activity patterns of C57Bl and C3H, as seen in their actograms, are, so to say, the inverted “mirror images” of their daily sleep profiles, as seen in their somnograms (Veasey et al., 2000).

In mice the decline of nighttime activity marks the onset of increased REM and NREM sleep episodes (Comai et al., 2013) and there are differences between the melatonin receptor KO animals. The MT1 signaling is involved in the modulation of the daily rhythm of REM sleep (Ochoa-Sanchez et al., 2011). EEG/EMG studies in MT1 KO mice showed a decrease in REM sleep during the light phase (when mice are mainly inactive) accompanied by an increase in the amount of NREM sleep (Comai et al., 2013). Melatonin promotes NREM sleep probably by binding to the MT2 receptors located reticular thalamic nucleus (Ochoa-Sanchez et al., 2011). Indeed, the above-mentioned EEG/EMG studies showed that in MT2 KOs NREM sleep is decreased during the light phase (when mice are inactive) paralleled by an increase in wakefulness (Comai et al., 2013). Interestingly in mice lacking both melatonin receptors an increase in wakefulness and a reduction in REM sleep occurred (Comai et al., 2013) suggesting that the melatoninergic system modulates wakefulness rather than sleep.

The time of sleep onset depends on the homeostatic sleep pressure which progressively accumulates during wakefulness and the circadian system (Borbély and Achermann, 1992; Dijk and Lockley, 2002). Adenosine plays an important role in the regulation of sleep homeostasis: adenosine levels accumulated during wakefulness increase throughout the brain and thereby increase sleepiness (Brown et al., 2012; Huang et al., 2014). The melatoninergic system might be involved in the increase of adenosine by acting on the ecto-5-nucleotidase, an enzyme which converts AMP to adenosine. Differences in the rhythmic ectonucleotidase mRNA expression between melatonin-proficient C3H and melatonin-deficient C57Bl mice are present in several brain structures (Homola et al., 2015). These differences in the rhythmic ectonucleotidase mRNA expression appeared to depend on the MT2 receptor subtype (Homola et al., 2016). The impact of the MT2 receptor on the elevation of ectonucleotidase RNA levels at night-time might be of relevance since this receptor is suggested to play an important role of in sleep regulation (Ochoa-Sanchez et al., 2011; Comai and Gobbi, 2014).

### Hippocampal Neuronal Plasticity and Behavior

The hippocampus plays an important role in the consolidation of information from short-term memory to long-term memory, as well as in spatial and temporal orientation that enable navigation. Here melatonin, by acting on melatonin receptors, modulates functional and structural neuronal plasticity, the basis for memory formation and learning. Functional hippocampal neuronal plasticity such as long-term potentiation, the persistent strengthening of synapses, has been shown to be inhibited by melatonin via the cAMP signaling pathway. This inhibitory effect of melatonin seems be mediated via the MT2 receptor, as the inhibitory effect on long-term potentiation was absent in both MT2 and MT1/MT2 double KO mice, but was present in MT1 receptor KO mice (Wang et al., 2005). Accordingly, MT1/MT2 double KO mice showed enhanced long-term potentiation responses and better memory test performances as compared to the melatonin-proficient C3H animals (O'Neal-Moffitt et al., 2014).

Moreover, structural hippocampal neuronal plasticity such as adult neurogenesis, is also modulated by melatonin. Specifically, C3H mice with functional MT1/MT2 receptors show a time-of-day-dependent fluctuation in the number of proliferation neuronal stem/progenitor cells in contrast to the MT1/MT2 double KO mice (Fredrich et al., 2017). Additional promoting effects of melatonin on adult neurogenesis such as antioxidative activity and enhanced expression of neurotrophic factors are suggested (reviewed in Ali and von Gall, 2022).

At the behavioral level, the MT2 receptor plays an important role for the beneficial action of chronic melatonin treatment on long- term object recognition memory, while the MT1 may mediate the effects of melatonin on object location memory (Pistono et al., 2021). Indeed, C3H mice showed better spatial learning efficiency than C3H mice lacking the MT1 and MT2 receptors (Jilg et al., 2019). Thus, the melatoninergic system shapes time-of-day-dependent learning efficiency and provides a time cue for hippocampal functions (Jilg et al., 2019). These data show the relevance of the melatoninergic system for cognitive performance, although the mechanisms still need to be unraveled.

Furthermore, the melatoninergic system seem to modulate emotion related behavior such as depressive- and anxiety-like behaviors. Melatonin-deficient C57Bl mice show a more pronounced depression-like behavior as compared to melatonin-proficient C3H mice in the force swim tests (Kurtuncu et al., 2005) while C3H mice expressed more anxiety-like behaviors than C57Bl animals in an open field experiment (Ennaceur et al., 2006), but not in a light/dark box or under a free exploratory paradigm (Kopp et al., 1999). This has been confirmed by investigations of MT receptor KO mice. MT1 KO mice showed an increased immobility in the forced swim and tail suspension test indicating depressive-like activity (Weil et al., 2006; Adamah-Biassi et al., 2014; Comai et al., 2015).

Furthermore, MT1 KO mice show changes in anxiety-related parameters (Adamah-Biassi et al., 2014; Comai et al., 2015) and decreased sucrose consumption, indicating anhedonia (Comai et al., 2015). Taken together, MT1 KO mice display many features that are core symptoms of human melancholic depression (Comai et al., 2015). Remarkably, the depressive-like symptoms could be reversed by chronic treatment with desipramine, a serotonin-reuptake inhibitor used to treat patients with depression (Comai et al., 2015). Overall the data suggest a modulatory role especially of the MT1 receptor in the neuronal networks for emotion-related behavior, although there is little evidence for an expression of this receptors in the respective brain regions. Importantly, (Comai et al., 2015), showed that MT1 KO mice have reduced time-of-day-dependent variations in serum levels of corticosterone and in the electrical activity of norepinephrine- and serotonin-neurons in the brain stem, which are involved in the pathophysiology of depression. This indicates that the changes in emotional behavior of MT1 KO mice are due to a disruption in the circadian system rather than to a change in the activity of isolated neuronal networks.

### Energy Metabolism and Glucose Homeostasis

Several metabolic diseases (i.e., diabetes type 2; obesity) are linked to the circadian system and in some of them the melatoninergic system seems to be involved (Stenvers et al., 2019). A close relationship between circadian rhythms with type 2 diabetes appears likely, since glucose metabolism displays circadian cycles (Jarett et al., 1972; Aparicio et al., 1974). Furthermore, circadian disruption leads to the development of type 2 diabetes (Coomans et al., 2013; Morris et al., 2016; Stenvers et al., 2019). A possible link between the melatoninergic system and glucose metabolism became evident relatively early, since melatonin-deficient C57Bl mice have a glucose-intolerant phenotype, probably due to an impaired glucose-stimulated insulin secretion, which is not found in melatonin-proficient C3H animals (Kaku et al., 1988). By now, it is known that both melatonin receptors are expressed in pancreatic islets in humans and rodents (Dubocovich et al., 2010; Nagorny et al., 2011). Studies in mice located the MT1 receptor in the pancreatic α-cells, while MT2 receptors were located in β-cells (Nagorny et al., 2011). These data indicate that the melatoninergic system is involved in insulin and glucagon secretion.

Furthermore, these melatonin receptor KO animals were used to determine the mechanisms by which these receptors contribute to regulation of glucose homeostasis and insulin sensitivity. The removal of both melatonin receptors abolishes the daily rhythm in blood glucose levels (Owino et al., 2016). Mice lacking the MT1 receptor exhibit higher mean blood glucose levels (Mühlbauer et al., 2009) and are more glucose intolerant and insulin resistant as compared to WT and MT2 KO male mice (Contreras-Alcantara et al., 2010). This systemic insulin resistance of MT1 KO mice is accompanied by an impaired skeletal muscle glucose uptake, adipose tissue glucose uptake and a significantly reduced liver insulin sensitivity (Owino et al., 2018). In line with these data several MT1 receptor variants were found that are associated with increased fasting plasma glucose levels and type 2 diabetes risk in humans (Bouatia-Naji et al., 2009; Prokopenko et al., 2009; for review see; Karamitri et al., 2013).

In pancreatic islets of MT1-; MT2- and double KO mice the insulin secretion was reduced (Mühlbauer et al., 2009) indicating an inhibitory role of the melatoninergic system on insulin secretion and/or synthesis. Also, the basal glucagon secretion was reduced in the pancreatic islets of mice lacking the MT2 receptor (Stumpf et al., 2008).

Inconsistent data on glucose metabolism were obtained in MT2 KO animals: one study reported that MT2 KO mice showed no specific phenotype as compared to the WT and are neither insulin resistant nor glucose intolerant (Contreras-Alcantara et al., 2010). Another more recent study reported a decreased hepatic insulin sensitivity and an increased insulin secretion in MT2 KO mice (Tuomi et al., 2016). However, these discrepancies might result from the different sexes used in these studies and/or strain differences. The earlier metabolic characterization of MT2 KO mice was conducted in male mice backcrossed with C3H/f^+/+^ to remove the rd mutation, whereas the more recent study was performed in female mice. Notably, variants of the MT2 receptor in humans have been linked to impairments in insulin secretion as well as increased fasting glucose levels (Bouatia-Naji et al., 2009; Lyssenko et al., 2009; Bonnefond et al., 2012; for review see; Karamitri et al., 2013).

There are further important metabolic effects of melatonin on other tissues, as well as effects on food uptake and timing. Leptin is an adipose tissue-derived hormone that is, released in a circadian manner from adipocytes. Leptin is involved in the regulation of energy balance by inhibiting *hunger*, which in turn diminishes fat storage in adipocytes.

MT1 KO mice have been shown to be leptin-resistant since the administration of exogenous leptin failed to induce the phosphorylation of signal transducers and activators of transcription 3 (STAT3) in the arcuate nucleus of these animals (Buonfiglio et al., 2019). Furthermore, the leptin receptor mRNA levels in the hypothalamus of MT1 KO were reduced as compared to controls (Buonfiglio et al., 2019). Therefore, the lack of MT1 signaling induces leptin resistance probably by down-regulation of the leptin receptor expression. Since leptin resistance results in an increased food intake and weight gain, the melatoninergic system might be associated with body weight control and diet induced obesity. Indeed, in a recent study which examined the effect of high fat diet, MT1 receptor KO mice displayed a higher cumulative weight gain and hyperglycemia as compared to their WT mice (Owino et al., 2019).

In addition, melatonin receptors seem to be involved in food intake and its timing. MT1 receptor KO mice spent more time feeding than C3H mice. However, the MT1 receptor deletion did not alter the amount of food ingested, but the temporal pattern of feeding compared to WT mice (Fischer et al., 2017).

In an experiment testing the rewarding/reinforcing properties of food, the MT1 receptor KO mice consumed less snack food as compared to MT2 receptor KO and C3H mice. In addition, the melatonin-proficient C3H conditioned to snack food during the light phase developed a place preference, whereas mice lacking both melatonin receptors did not develop a place preference for snack food (Clough et al., 2018). This suggests that the melatoninergic system may also modulate the reward pathway.

### Materno-Fetal Communication

As shown for various species, the melatoninergic system plays an important role in maternal-fetal communication by providing rhythmic signals to the fetuses who are not yet able to produce melatonin. Fetuses get access to maternal melatonin via the placenta and newborn animals via the milk (Weaver and Reppert, 1986; Weaver et al., 1987; Mendez et al., 2012; Bates and Herzog, 2020; Lužná et al., 2021). Mouse models were also used to investigate maternal-fetal communication (Čečmanová et al., 2019), however only very few studies in mice relate to the melatoninergic system (Ansari et al., 2009; Christ et al., 2012). These data provided evidence that maternal melatonin is an important synchronizing signal for circadian rhythms *in utero* and postnatally. However, as many mouse strains are melatonin-deficient, melatonin seems to be largely dispensable for the development of the circadian system.

## Conclusion

The comparison between mouse strains with an intact and a compromised melatoninergic system have proven useful to understand the physiological impact of the melatoninergic system. These investigations provide a guideline for future investigations on the impact of the melatoninergic system on various diseases. The recent development of melatonin-proficient congenic mice on a C57Bl-background offers opportunities for background-independent comparisons, yet a melatonin-deficient C3H-mouse is a another desiderate that would—in the interplay with the MT receptor KOs that are available for that strain—help to further dissect the functional roles of the components of the system.
